# Adaptability of minimally invasive thoracoscopic repair for congenital diaphragmatic hernia: a neonatal case report from a low-income country

**DOI:** 10.1097/RC9.0000000000000868

**Published:** 2026-08-19

**Authors:** Mohammad Tareq Rahimi, Abdulwahab Amanat, Mamonullah Asmati, Nawaz Sharif, Tawhid Abdulrahman Rahimi, Mohammad Hussain Mohammadi

**Affiliations:** a Department of Pediatric Surgery, French Medical Institute for Mothers and Children, Kabul, Afghanistan; b Department of Medicine, Cheragh Medical Institute, Kabul, Afghanistan; c Department of Medicine, Alberoni University, Kapisa, Afghanistan

**Keywords:** Bochdalek hernia, congenital diaphragmatic hernia, diaphragm abnormalities, minimally invasive surgical procedures, neonate, pediatric surgery, thoracoscopic repair

## Abstract

**Background:**

Congenital diaphragmatic hernia (CDH) is a rare but potentially life-threatening congenital anomaly characterized by herniation of abdominal organs into the thoracic cavity, resulting in respiratory compromise and pulmonary hypoplasia.

**Case presentation:**

A 2-day-old female neonate weighing 2.5 kg presented with severe respiratory distress, cyanosis, and a scaphoid abdomen. Chest radiography and computed tomography revealed a large left-sided Bochdalek hernia containing the stomach, spleen, left kidney, and bowel loops, causing compression of the left lung and mediastinal shift. After stabilization with mechanical ventilation, the patient underwent thoracoscopic repair. The patient was positioned in the right lateral decubitus position, and three 5-mm ports were inserted. Carbon dioxide insufflation was maintained at 5 mmHg with a flow rate of 1 L/min. The herniated viscera were reduced, and a 5 × 5 cm diaphragmatic defect was closed primarily with interrupted 3-0 polypropylene sutures, without mesh reinforcement. The operative time from skin incision to wound closure was 35 minutes. The postoperative course was uneventful, with extubation on the first postoperative day and discharge on the fourth postoperative day. No recurrence was observed over 1 year of follow-up.

**Conclusion:**

This case demonstrates the feasibility of thoracoscopic primary repair of CDH in a carefully selected neonate within a resource-limited setting, resulting in favorable short- and long-term outcomes.

## Introduction

Congenital diaphragmatic hernia (CDH) is a rare developmental anomaly resulting from incomplete formation of the diaphragm during fetal life, with an estimated incidence of 1–4 per 10 000 live births^[^1^]^. The defect allows abdominal viscera to herniate into the thoracic cavity, impairing normal lung development and potentially leading to pulmonary hypoplasia and pulmonary hypertension, which remain the principal determinants of morbidity and mortality^[^2^]^. Despite advances in prenatal diagnosis, neonatal intensive care, and surgical management, CDH continues to represent a significant challenge in neonatal surgery^[^3^]^.

Bochdalek hernia, a posterolateral diaphragmatic defect, accounts for approximately 70–75% of CDH cases and most commonly occurs on the left side^[^4^]^, while Morgagni hernia represents 20–25%. Central defects occur in 2–5%, and bilateral cases are rare with a poor prognosis^[^1,5^]^. Clinical manifestations vary according to the degree of pulmonary compromise and may include respiratory distress, cyanosis, diminished breath sounds, mediastinal shift, and a scaphoid abdomen during the neonatal period. Early diagnosis and appropriate perioperative management are essential for improving outcomes^[^2^]^.

HIGHLIGHTS

- Thoracoscopic repair was successfully performed in a neonate with congenital diaphragmatic hernia.
- A large 5 × 5 cm defect was closed without mesh.
- Multiple abdominal organs were reduced thoracoscopically.
- Recovery was uneventful, with no recurrence at 1 year.
- Advanced neonatal minimally invasive surgery was feasible in a resource-limited setting.

Surgical repair remains the definitive treatment for CDH. Traditionally, repair has been performed through laparotomy or thoracotomy; however, minimally invasive techniques have gained increasing acceptance in selected neonates^[^6,7^]^. Thoracoscopic repair offers several potential advantages, including reduced postoperative pain, shorter hospital stays, avoidance of thoracotomy-related musculoskeletal complications, and improved cosmetic outcomes^[^7^]^. Nevertheless, concerns remain regarding recurrence rates, patient selection, and the technical challenges associated with neonatal minimally invasive surgery, particularly in resource-limited environments^[^3^]^.

Reports describing neonatal thoracoscopic repair of CDH from low-income countries remain limited. We present a case of a 2-day-old neonate with a large left-sided Bochdalek hernia containing the stomach, spleen, left kidney, and bowel loops that was successfully managed by thoracoscopic primary repair without mesh reinforcement. This report highlights the feasibility of minimally invasive repair in a carefully selected patient within a resource-constrained setting and is presented in accordance with the SCARE 2025 guidelines^[^8^]^.

## Case presentation

**Figure 1.**
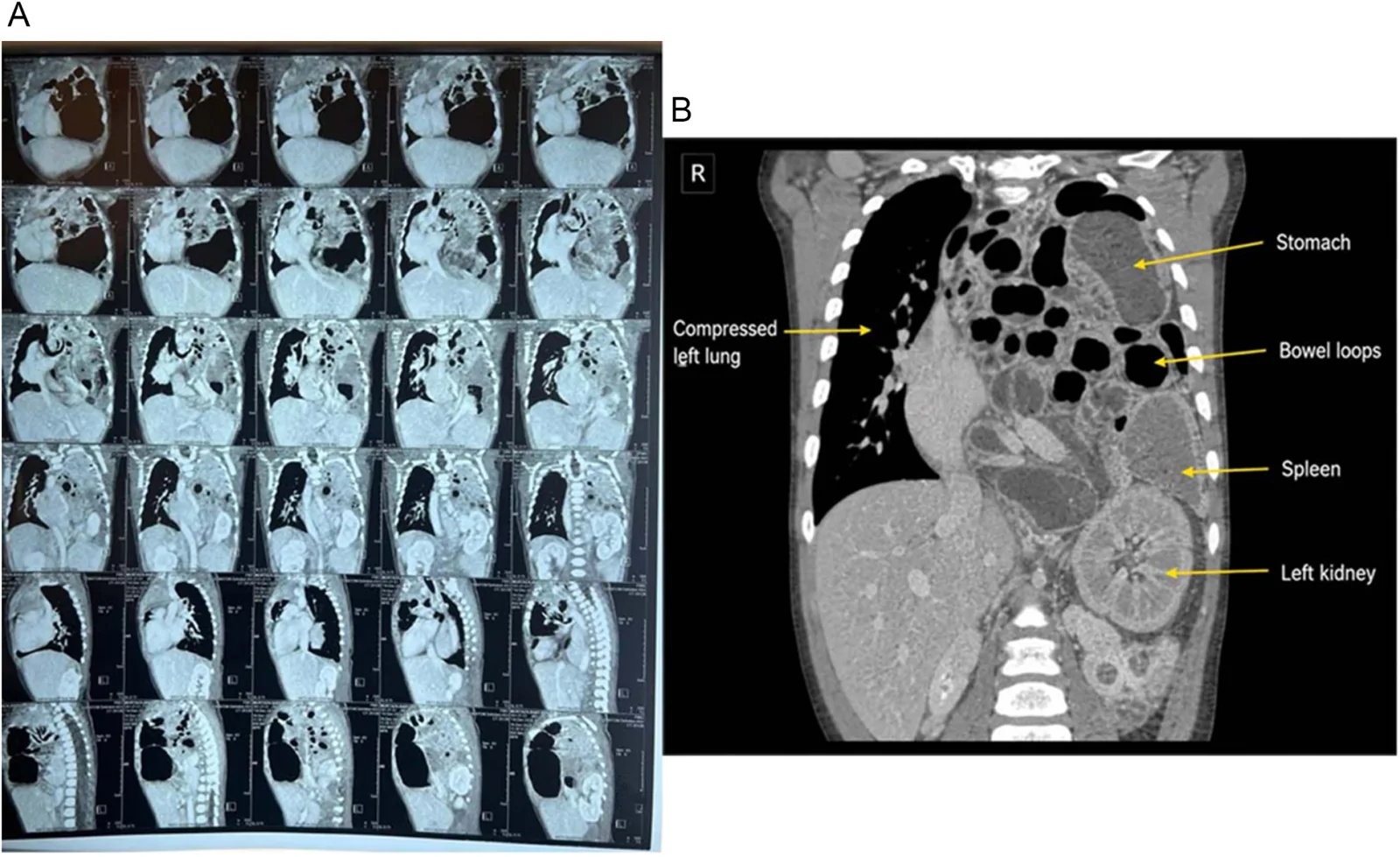
(A) Computed tomography (CT) of the thorax and abdomen demonstrates an extensive left-sided Bochdalek hernia. Multiple abdominal organs, including the stomach, spleen, kidney, and bowel loops, are herniated into the left hemithorax. The displaced viscera cause marked compression of the ipsilateral lung and a mediastinal shift to the right. (B) Coronal CT image shows a left-sided diaphragmatic hernia with herniation of the stomach and bowel loops into the thoracic cavity, resulting in marked compression of the left lung. The spleen and left kidney are identified for anatomical reference.

A 2-day-old term female neonate weighing 2.5 kg was admitted to the Neonatal Intensive Care Unit with severe respiratory distress, central cyanosis, and inability to breastfeed. Examination revealed marked subcostal and intercostal retractions, diminished breath sounds over the left hemithorax, rightward displacement of the cardiac impulse, and a scaphoid abdomen. Echocardiography excluded structural cardiac malformations. Chest radiography and computed tomography confirmed a large left-sided Bochdalek CDH with herniation of the stomach, spleen, left kidney, and bowel loops, compressing the left lung and displacing the mediastinum (Fig. 1).

Laboratory studies showed polycythemia (hemoglobin 20.1 g/dL, hematocrit 56.9%) and mild coagulopathy (International Normalized Ratio 1.7, a prolonged activated Partial Thromboplastin Time). Intramuscular vitamin K (1 mg) corrected the abnormality, and fresh frozen plasma was not required. After stabilization with mechanical ventilation and with informed parental consent, thoracoscopic repair was undertaken.

**Figure 2.**
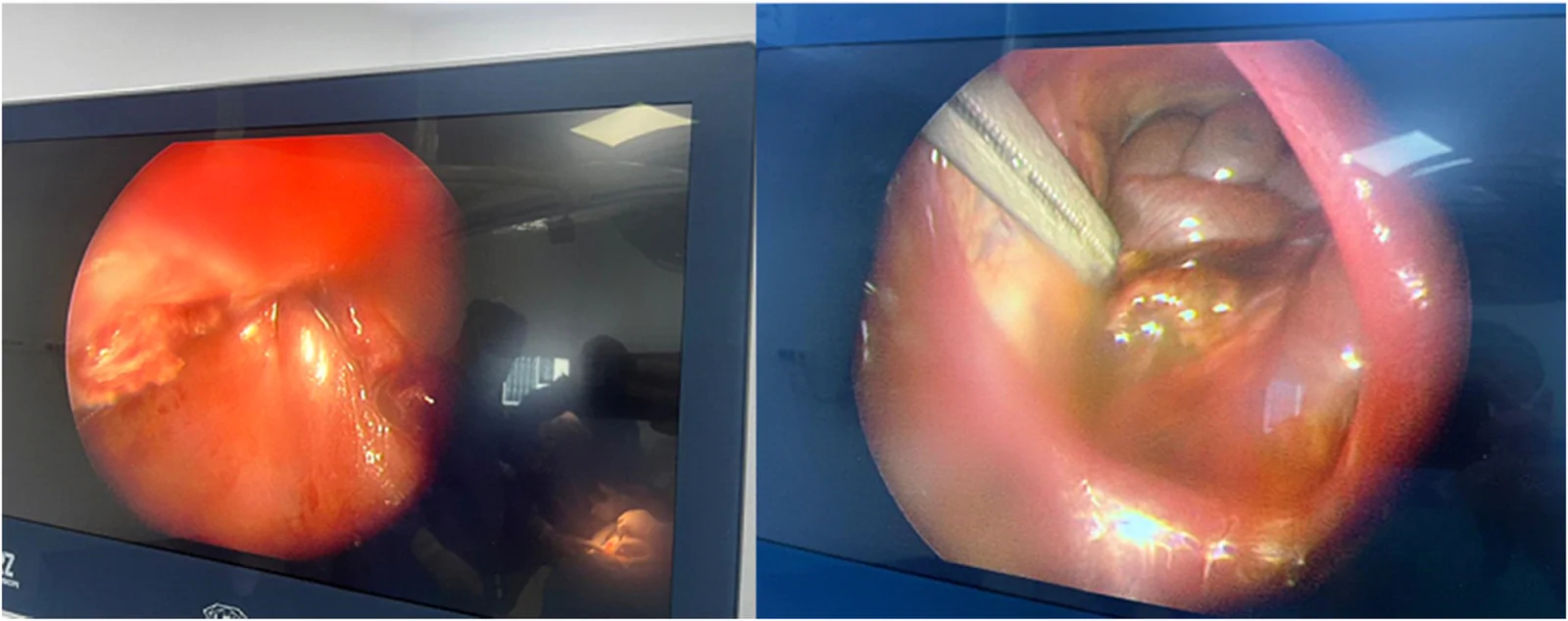
Thoracoscopic intraoperative view of the diaphragmatic defect with herniated abdominal viscera prior to reduction.

On day two of life, thoracoscopic repair was performed under general anesthesia in the right lateral decubitus position. Three 5-mm trocars were placed, and carbon dioxide insufflation (5 mmHg, 1 L/min) facilitated the reduction of herniated viscera with Babcock forceps. A posterolateral diaphragmatic defect measuring 5 × 5 cm (CDH Study Group Type B) was identified. Adequate native tissue permitted tension-free primary closure with four to five interrupted 3-0 polypropylene sutures. Mesh reinforcement was unnecessary, and a chest drain was not required. Operative time was 35 minutes (Fig. 2).

**Figure 3.**
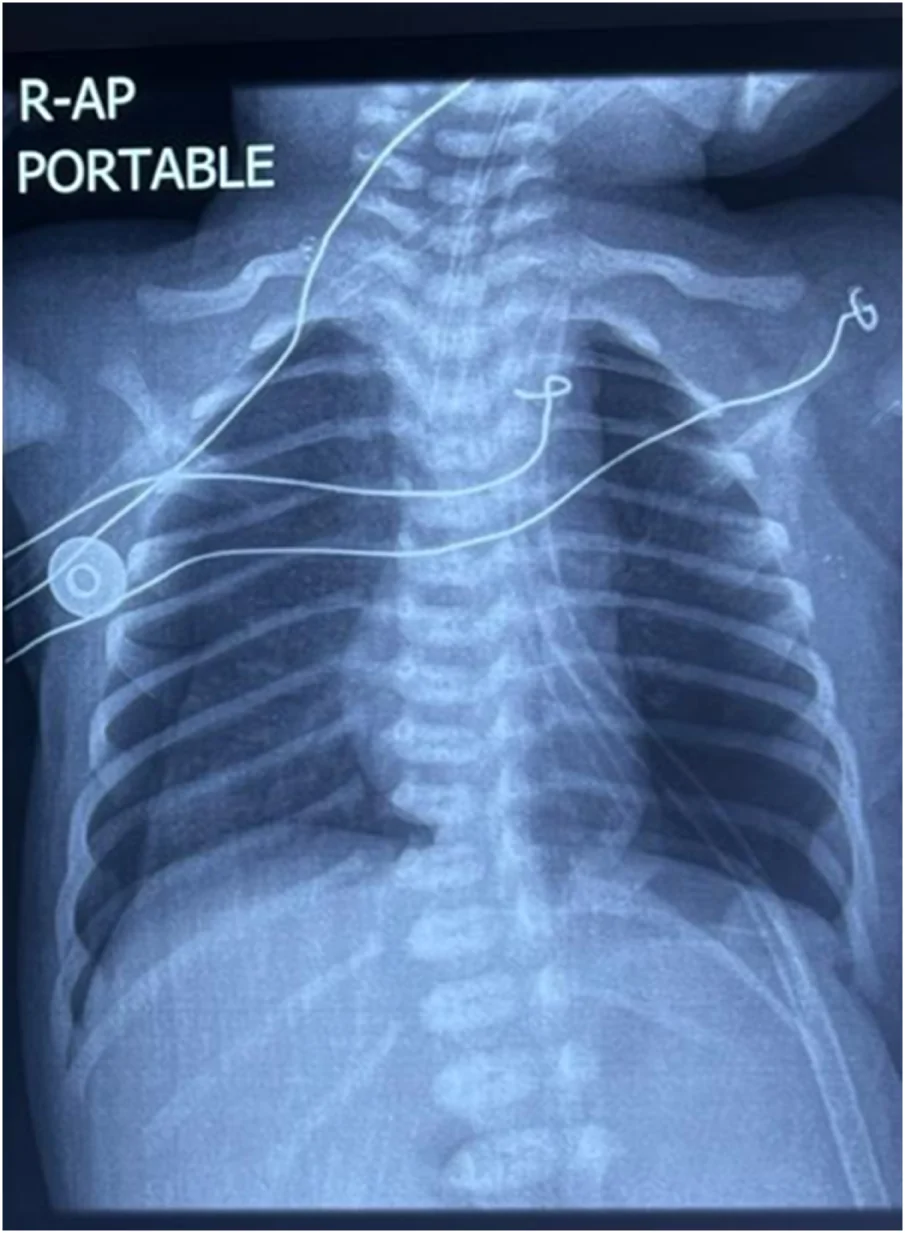
A follow-up portable chest radiograph shows a stable repair with no evidence of recurrence.

The patient was extubated on postoperative day 1, resumed enteral feeding, and improved steadily. She was discharged on day 4 in stable condition. Follow-up during infancy showed normal growth and development without respiratory symptoms. At 1 year, chest radiography confirmed no recurrence, and the child remained clinically well with excellent cosmetic results (Fig. 3).

## Discussion

CDH remains a challenging neonatal surgical condition because outcomes are largely determined by the severity of pulmonary hypoplasia, pulmonary hypertension, associated anomalies, and perioperative management^[^1^]^. Consistent with previous reports, our patient presented during the early neonatal period with severe respiratory distress, cyanosis, diminished breath sounds over the affected hemithorax, and a scaphoid abdomen. Computed tomography confirmed a large left-sided Bochdalek hernia containing the stomach, spleen, left kidney, and bowel loops, resulting in significant compression of the left lung and a mediastinal shift^[^9,10^]^.

Surgical repair remains the definitive treatment for CDH. Although open repair through laparotomy or thoracotomy has traditionally been considered the standard approach, minimally invasive surgery has increasingly been adopted in selected neonates and infants^[^3^]^. Thoracoscopic repair offers several advantages, including enhanced visualization of the diaphragmatic defect, reduced postoperative pain, shorter recovery, avoidance of thoracotomy-related musculoskeletal deformities, and improved cosmetic outcomes^[^11^]^. Successful thoracoscopic repair has been reported even in very-low-weight neonates, demonstrating the feasibility of this approach in carefully selected patients^[^12^]^.

Despite these advantages, concerns regarding recurrence remain an important consideration. Recent systematic reviews and meta-analyses have demonstrated that thoracoscopic repair may be associated with higher recurrence rates than open repair, particularly in large defects requiring prosthetic patch reconstruction^[^13^]^. Current clinical guidelines therefore emphasize careful patient selection, surgeon experience, and meticulous repair technique when considering a minimally invasive approach^[^3^]^.

In this case, thoracoscopic repair was chosen because the patient achieved adequate preoperative cardiopulmonary stabilization, and the herniated contents of the stomach, spleen, bowel loops, and left kidney could be safely reduced thoracoscopically. Carbon dioxide insufflation facilitated the gentle reduction of the spleen and bowel into the abdomen, minimizing tissue manipulation. In contrast, significant liver herniation often makes thoracoscopic reduction technically challenging and may favor an abdominal approach via laparotomy.

The principal intraoperative challenge was primary closure of the relatively large diaphragmatic defect. Wide defects may increase closure tension and are occasionally associated with additional intra-abdominal anomalies. Nevertheless, adequate native diaphragmatic tissue was present in this patient, allowing tension-free primary repair without prosthetic reinforcement. Defect size is an important predictor of outcomes and recurrence, with larger defects frequently associated with increased morbidity and poorer pulmonary outcomes^[^14^]^. Whenever feasible, primary repair is generally preferred over prosthetic reconstruction because patch repairs may be associated with recurrence and long-term complications related to growth and tissue integration.

An additional technical observation in this case was the role of low-pressure carbon dioxide insufflation in facilitating reduction of the herniated viscera. Thoracoscopic insufflation created an adequate working space and assisted gradual reduction of the stomach, spleen, kidney, and bowel loops into the abdominal cavity with minimal manipulation. Following complete reduction, closure of the defect required only a limited number of interrupted sutures. Although the operative time was 35 minutes from skin incision to wound closure, the relatively short duration likely reflected favorable anatomy, the feasibility of primary closure without patch reconstruction, and the experience of the surgical team. Similar technical refinements have been described in recent thoracoscopic series, emphasizing that surgical outcomes are more closely related to patient selection and repair quality than operative duration alone^[^15^]^.

Thoracoscopic neonatal surgery is infrequently reported in low-income countries because of limitations in specialized equipment, pediatric anesthesia expertise, and neonatal intensive care resources. Reports from India, sub-Saharan Africa, and other resource-constrained regions have highlighted the challenges associated with advanced neonatal surgical care^[^16,17^]^. Although Afghanistan is a resource-limited country, this procedure was performed in a tertiary pediatric referral center with dedicated pediatric surgery and pediatric anesthesia services. Therefore, the successful outcome observed in this case should not be interpreted as evidence that thoracoscopic repair is universally applicable to all centers but rather demonstrates that advanced minimally invasive neonatal surgery can be safely implemented when appropriate expertise and infrastructure are available.

The patient remained asymptomatic over 1 year of follow-up, with no clinical or radiological evidence of recurrence. Long-term surveillance remains important because recurrence may occur months or years after repair, particularly in patients with larger defects^[^18,19^]^.

Successful thoracoscopic repair of CDH requires both surgical expertise and adequate postoperative intensive care. Key resources include neonatal ventilation, continuous monitoring, arterial blood gas analysis, skilled nursing, pediatric anesthesia, and timely radiologic support. These capabilities are fundamental to the safe implementation of minimally invasive neonatal surgery in resource-constrained settings.

## Conclusion

CDH remains a life-threatening neonatal condition that requires prompt diagnosis, stabilization, and timely surgery. This case shows that thoracoscopic primary repair can be safely performed in selected neonates within resource-limited settings when surgical expertise, pediatric anesthesia, and intensive care support are available. Despite a large 5 × 5 cm defect, tension-free closure without mesh achieved an excellent outcome with no recurrence at 1 year. Careful patient selection based on cardiopulmonary stability is essential, and larger studies are needed to assess long-term outcomes.

## Data Availability

Not applicable.
